# The duration aftereffect does not reflect adaptation to perceived duration

**DOI:** 10.1371/journal.pone.0213163

**Published:** 2019-03-04

**Authors:** Jim Maarseveen, Chris L. E. Paffen, Frans A. J. Verstraten, Hinze Hogendoorn

**Affiliations:** 1 Utrecht University, Helmholtz Institute, Department of Experimental Psychology, Utrecht, The Netherlands; 2 The University of Sydney, School of Psychology, Brain and Mind Centre, Sydney, NSW, Australia; 3 The University of Melbourne, Melbourne School of Psychological Sciences, Melbourne, VIC, Australia; Brandenburgische Technische Universitat Cottbus-Senftenberg, GERMANY

## Abstract

Recent studies have provided evidence for a role of duration-tuned channels in the encoding of duration. Duration encoding in these channels is thought to reflect the time between responses to the onset and offset of an event. This notion is in apparent conflict with studies that demonstrate that the perceived duration of an event can vary independently from the time separating its perceived onset and offset. Instead, these studies suggest that duration encoding is sensitive to other temporal aspects of a sensory event. In the current study, we investigated whether duration-tuned channels encode duration based on the time between the on- and offset of an event (onset-offset duration), or if they encode a duration corresponding to the perceived duration of that event. We used a duration illusion to dissociate onset-offset duration and perceived duration and measured whether repeated exposure to illusion-inducing stimuli caused adaptation to the onset-offset duration or the perceived duration of these illusion-inducing stimuli. We report clear evidence for adaptation to the onset-offset duration of illusion-inducing stimuli. This finding supports the notion that duration-tuned mechanisms respond to the time between the onset and offset of an event, without necessarily reflecting the duration perceived, and eventually reported by the participant. Implications for the duration channel model and the mechanisms underlying duration illusions are discussed.

## Introduction

Recently, it has been proposed that duration-tuned mechanisms underlie the encoding of duration [1,2]. According to this idea, the brain contains groups of duration-tuned neurons that respond selectively to specific ranges of durations. Summation of the population response of these groups of duration-tuned bandpass-neurons (or channels) allows for implicit temporal signals to be transformed into an explicit code for duration that is both accurate and reliable. This explicit signal can then be stored, manipulated, and used to guide subsequent behavior [1].

The proposal for channel-based encoding of duration is very similar to the mechanisms that are thought to underlie the encoding of a range of other sensory features such as orientation [3,4], motion direction [5,6], pitch [7], and numerosity [8,9]. Similar to these other stimulus features, support for the channel-based encoding of duration comes from studies demonstrating duration tuning in both behavioral and neuronal responses [1,10]. For example, several studies have shown that adaptation to duration leads to a duration after-effect (DAE) for subsequently presented durations [1,11–15]. More specifically, these studies demonstrate that adaptation to a particular duration in one modality causes the perceived duration of subsequent durations in that same modality to shift away from the adapted duration. For example, after adapting to a visual event lasting 400 ms, the perceived duration of visual events with a shorter duration (i.e. 200 ms) will decrease, while the perceived duration of events with a longer duration (i.e. 800 ms) will increase. These results are taken to reflect selective adaptation of duration-tuned neurons, resulting in a shifted population response for durations close to the adapted duration [1,16]. Similarly, studies have shown that training observers to discriminate durations leads to increased performance on the trained but not the untrained durations [17]. In line with the adaptation results, these results suggest channel-specific training benefits and are similar to the results observed for discrimination training in other features encoded in a channel-based fashion [17,18]. Furthermore, a recent fMRI study using fMRI adaptation demonstrated a decreased BOLD-response in the (right) inferior parietal lobule (IPL) following repetitions of identical duration [10]. This fMRI adaptation did not occur when the two durations were different, indicating that the BOLD-responses in this area reflected selective responses to specific durations.

These findings support the notion that selectively tuned channels underlie the encoding of duration in a way that is similar to the encoding of other sensory properties. However, it is not clear what aspect(s) of a sensory event these channels are responding to. According to one idea, duration channels are sensitive to the temporal distance separating the neural responses to the onset and offset of a sensory event. In other words, duration tuned channels are thought to respond differentially to the offset of an event depending on the time since the response to the onset of that same event [1]. Evidence for these onset-dependent offset responses comes from animal physiology studies demonstrating duration-tuned responses in single cells in both Brown bat auditory cortex [19,20], cat auditory cortex [21] and cat visual cortex [22].

However, this idea of encoding duration based on the temporal distance between the onset and offset responses is in apparent contrast with the fact that the perceived duration of an event can be manipulated, without any concurrent changes in our perception of the onset and offset of that same event [23,24]. For example, in the Temporal Frequency Induced Time Dilation (TFITD) illusion, increasing the speed or temporal frequency of an event increases its perceived duration without affecting the perceived onset and offset of that same event [24]. Assuming that the duration encoded by the duration channels informs our perception of duration, we would expect a strong relation between the temporal distance separating the perceived moments of onset and offset, and the duration perceived by the observer. As such, this dissociation between the separation of perceived onset and offset on the one hand, and perceived duration on the other, seems in contrast with the idea that duration channels extract duration based solely on the temporal distance between onsets and offsets of the sensory signal.

To address this contrast, we used duration adaptation to probe the duration-tuned channels and adapted observers to a TFITD-inducing stimulus. By doing so, it is possible to dissociate between adaptation to the temporal distance separating the onset and offset of the sensory signal (onset-offset duration) and adaptation to a duration corresponding to the duration perceived by the observer. Participants adapted to repetitions of one of three stimuli: a rotating radial grating (the illusion-inducing stimulus); to a static radial grating matched to the temporal distance between the onset and offset of the illusion-inducing stimulus (the onset-offset matched stimulus); or to a static radial grating matched to the perceived duration of the illusion-inducing stimulus (the perceptually matched stimulus). Next, we measured whether the resulting DAE for the illusion-inducing stimulus reflected adaptation to either its onset-offset duration or its perceived duration. If participants adapt to the onset-offset duration of the illusion-inducing stimulus, the resulting DAE should match the DAE following adaptation to the onset-offset matched stimulus. However, if participants adapt to the perceived duration of an event, the DAE for the illusion-inducing stimulus should match the DAE for the perceptually matched stimulus.

## Methods

### Participants

25 Observers participated in this experiment. Of these 25, 5 participants were excluded after an initial measurement because they did not display a sufficiently large TFITD illusion (time dilation < 33%). Excluded participants completed an unrelated experiment. Details about the exclusion procedure can be found in the ‘TFITD illusion magnitude estimation’ section under Procedure. The remaining 20 participants completed the experiment (10 male, M_age_ = 24.30, SD = 4.73). All participants were naïve as to the purpose of the experiment and had normal or corrected-to-normal vision. Written informed consent was obtained before the experiment began. Following participation, participants received course credits or monetary compensation. This study was approved by the Ethics Committee at the Faculty of Social and Behavioral Sciences of Utrecht University and was conducted in accordance with the Declaration of Helsinki.

### Materials & stimuli

All stimuli were presented on a linearized 22-inch CRT monitor (1024 x 768 pixels, 100 Hz refresh rate) controlled by an Apple Mac Mini. Stimulus presentation was controlled using MATLAB 2015b (MathWorks, Inc.) and the Psychophysics Toolbox (Brainard, 1997; Pelli, 1997). Participants viewed the stimuli with their head resting in a chinrest placed 57 cm from the screen. The auditory stimuli were presented using a Sennheiser HD201 on-ear headset. All stimulus timings were verified using a dual-channel oscilloscope.

All stimuli consisted of a circular radial grating (diameter of 2.0°) with a sinusoidal luminance modulation (100% Michelson contrast) of 4 cycles. On each presentation, this stimulus was either static or rotating at 2.08 cycles/second. This resulted in each individual point of the stimulus being modulated sinusoidally at 8.33 Hz. All stimuli were presented on a gray background (32.5 cd/m^**2**^) and accompanied by a central fixation cross (64.3 cd/m^**2**^). Each radial grating was presented peripherally at a distance of 4° degrees from the central fixation cross. In all tasks, test and reference stimuli were presented at one of four possible positions, the location of which was counterbalanced across participants (0, 90, 180, or 270° angle from fixation). All adaptation stimuli were presented randomly across 10 possible locations at a 45 to 315° angle from the test stimulus with an inter stimulus interval (ISI) ranging between 500–750 ms. This spatial configuration was used to reduce the overall adaptation to the non-temporal stimulus features and the temporal frequency of our adaptation stimuli. In addition, this configuration resulted in a minimum separation of 3.06° of visual angle between the adaptation and test stimuli. At this distance any adaptation to the non-temporal features or temporal frequency of the adaptation stimuli should not affect our measurement of perceived duration at the test location [23,25], while still allowing for effective measurement of the DAE [14]. Auditory reference stimuli consisted of a burst of white noise (60dB) with a 10 ms linear onset and offset ramp.

### Procedure

#### TFITD illusion magnitude estimation

Since each participant differs in the magnitude of his or her TFITD illusion, the duration of the perceptually matched stimuli and the central reference stimulus needs to be tailored to each individual participant. We estimated the magnitude of the TFITD illusion for individual participants by having them completed a visual duration judgment task. In this task, participants compared the duration of a reference grating that lasted 300 ms. to a static test grating with a variable duration. Depending on the condition, the reference grating was either static or rotating. The order in which the reference and test were presented was randomized and counterbalanced across trials. For all presentations, we used an ISI jittered between 500–750 ms. The point of subjective equality (PSE) for each condition (static, rotating) was acquired over the course of 80 trials by varying the duration of the test stimulus using an accelerated stochastic approximation (ASA;[26]) set to converge to the 50% correct point. The ASA staircase aims to effectively converge on a selected response probability by quickly reducing step size. As a result, relatively few trials are required to produce a reliable estimate of the selected threshold. After participants completed this task, we used the PSEs estimated by each staircase to calculate an illusion factor (PSE_static_ / PSE_rotating_). We then multiplied this factor by the duration of the onset-offset matched stimulus (300 ms), to acquire the duration of the perceptually matched stimulus for the individual participant (M = 572.60 ms, SD = 129.00).

The current design relies on comparing a single critical condition (adaptation to illusion-inducing stimuli) to two ‘baseline’ conditions (adaptation to onset-offset matched stimuli & perceptually matched stimuli). As such, it is necessary to be able to dissociate between adaptation to the onset-offset matched baseline and the perceptually matched baseline. In other words, our design requires an observable difference in the PSEs following adaptation to the onset-offset matched and perceptually matched stimuli. The magnitude of the DAE depends strongly on the temporal distance between the adaption durations and the reference duration [1]. However, in the current study the temporal distance between adaptation durations and the reference durations is contingent on the magnitude of each individuals TFITD illusion. As such, it is problematic when participants do not display a significant TFITD illusion, as they do not provide data that contributes to answering our research question. To address this issue, we set an inclusion criterion at a minimum illusion magnitude of 33.33% (100 ms). By using this criterium, we aimed to optimize the inclusion of participants, while still obtaining a measurable DAE between our two ‘baseline’ conditions (onset-offset matched, perceptually matched). Participants who did not meet the inclusion criterion were excluded from the experiment and completed an unrelated experiment instead. As, mentioned above, five participants who had signed up were excluded based on this criterion. The remaining 20 participants completed the main adaptation experiment.

Next, we set the individual reference duration by calculating the logarithmic midpoint between the durations of the onset-offset matched (300 ms) and perceptually matched stimuli (M = 572.60 ms). This logarithmic midpoint (M = 412.09 ms, SD = 45.43) was used as the visual reference duration against which the DAE for each of the adaptation conditions was measured. Earlier work suggests that duration selective mechanisms show logarithmic scaling in preferred duration and sensitivity [1,21,22,27]. By using the logarithmic midpoint, we assure equal (perceptual) distance from the reference duration to the adaptation durations in the onset-offset matched and perceptually matched stimulus conditions. This procedure is similar to those used in earlier experiments, and allows us to measure the DAE as opposite shifts in perceived duration using the same range of test stimuli [11,14,15].

#### Auditory reference calibration

Our experiment employed a cross-modal duration judgment task in which the auditory reference duration always preceded the visual test stimulus. This procedure typically leads to a time order error [28] where, on average, the auditory reference will be perceived as having a longer duration than its visual counterpart with the same duration. To account for this, we set the duration of the auditory reference stimulus individually for each participant so that the perceived duration of the auditory stimulus was perceptually equivalent to that individual’s visual reference duration. To acquire each individual auditory reference duration, participants completed a cross modal duration judgment task in which they compared the duration of a static reference grating to the duration of an auditory test stimulus. For each individual participant, the duration for the static reference grating was based on the individual reference duration calculated in the previous task. The duration of the auditory test stimulus was varied using an ASA staircase set to converge at the 50% correct point over the course of 60 trials. The resulting estimates of the Point of Subjective Equality (PSE) were used to set the duration of the auditory reference used in the adaptation experiment (M = 370.63 ms, SD = 149.75).

#### Adaptation experiment

In separate blocks, participants adapted to 100 repetitions of one of three stimuli: 1) The illusion-inducing stimulus, which consisted of a rotating radial grating that lasted 300 ms. 2) The onset-offset matched stimulus, which consisted of a static radial grating that was matched to the onset-offset duration of the illusion-inducing stimulus (300 ms). 3) The perceptually matched stimulus, which consisted of a static radial grating matched to the perceived duration of the illusion-inducing stimulus. The duration of the perceptually matched stimulus was set individually for each participant (M = 572.60 ms,). During adaptation, participants did not perform any task and were instructed to maintain fixation on the central fixation cross.

**Fig 1:**
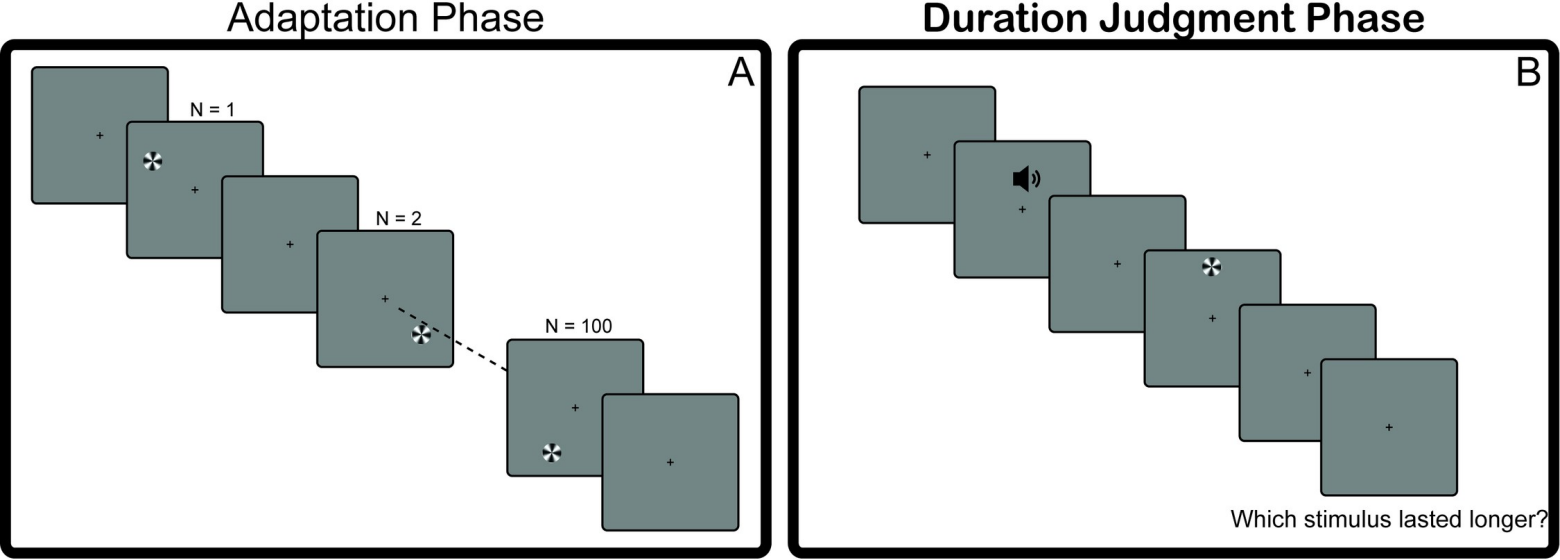
An overview of the experimental procedure for the adaptation experiment. Adaptation Phase (A): participants adapted to 100 repetitions of one of three adaptation stimuli: an illusion-inducing rotating radial grating (300 ms, 8.33Hz), a static radial grating matched to the onset-offset duration of the illusion-inducing stimulus (300 ms, static), or a static grating matched to the perceived duration of the illusion-inducing stimulus (M_duration_ = 572.60, static). Duration Judgment Phase (B): participants completed a duration judgment task in which they compared the duration of an auditory reference to that of a static visual test stimulus.

Each adaptation phase was followed by a test phase in which participants completed a cross-modal duration judgment task. Each duration judgment started with 4 top-up repetitions of the adaptation stimulus, followed by the auditory reference and a static visual test stimulus (ISI = 500–750 ms). The participant’s task was to indicate which of the reference—test pair had a longer duration (Fig 1). The duration of the test stimulus was varied using a Minimum Expected Entropy Staircase (MEES;[29]). This staircase allows for effective estimation of the PSE while still varying the duration of the stimuli presented to the participants. This variance in the duration of the test stimulus helps assure that participants pay attention to the task. In each block, participants completed 30 trials. In total, participants completed 9 blocks accounting to a total of 270 trials. This corresponded with participants completing a total of 90 trials per staircase, one for each adaptation condition. The entire experiment lasted about 2 hours and included a 15-minute break.

### Analysis

We calculated the PSE for each of the three adaptation conditions for each participant, by fitting a psychometric function using a logistic regression. The resulting PSEs correspond to the duration of the visual test stimulus that was perceived as being equal to the auditory reference duration. As such, higher PSEs indicate lower perceived durations of the test stimuli, while lower PSEs indicate higher perceived durations of the test stimuli.

All data were analyzed using Bayesian analysis using the open source statistics program JASP [30]. For all analyses, a common uninformative prior (Cauchy prior with width .707) was used. In Bayesian analysis a Bayes factor (BF) is used to express the relative probability that the current data were collected under one hypothesis (i.e. H_a_) vs. the probability that the data were collected under another hypothesis (i.e. H_0_). This relative evidence provided by the Bayes factor can then be evaluated using a common rule under which a BF_10_ > 3 is taken as evidence in favor of H_a_ and BF_10_ < 1/3 as evidence in favor of H_0_ [31,32]. It is important to note here that while higher/lower BFs indicate higher relative evidence for one model over another, it does not indicate a larger effect size. To judge the size of each effect, we encourage the reader to evaluate the provided figure as well as the averages and standard deviations reported for each statistical test.

One of the advantages of a Bayesian approach is that it allows to quantify the evidence for both our H_a_’s (the results from two conditions are different), as well as for our H_0_’s (the results from two conditions do not differ). This advantage over inferential statistics is critical in our current design. Our main hypothesis resolves around the notion that the DAE following adaptation to illusion inducing stimuli will reflect either adaptation to the onset-offset duration of the perceptually matched duration of that stimulus. In other words, we expect that the DAE following adaptation to illusion inducing stimuli will not differ from the DAE found in either the onset-offset matched or perceptually matched stimulus conditions. Quantification of evidence for the H_0_ allows us to test this hypothesis appropriately.

## Results

**Fig 2:**
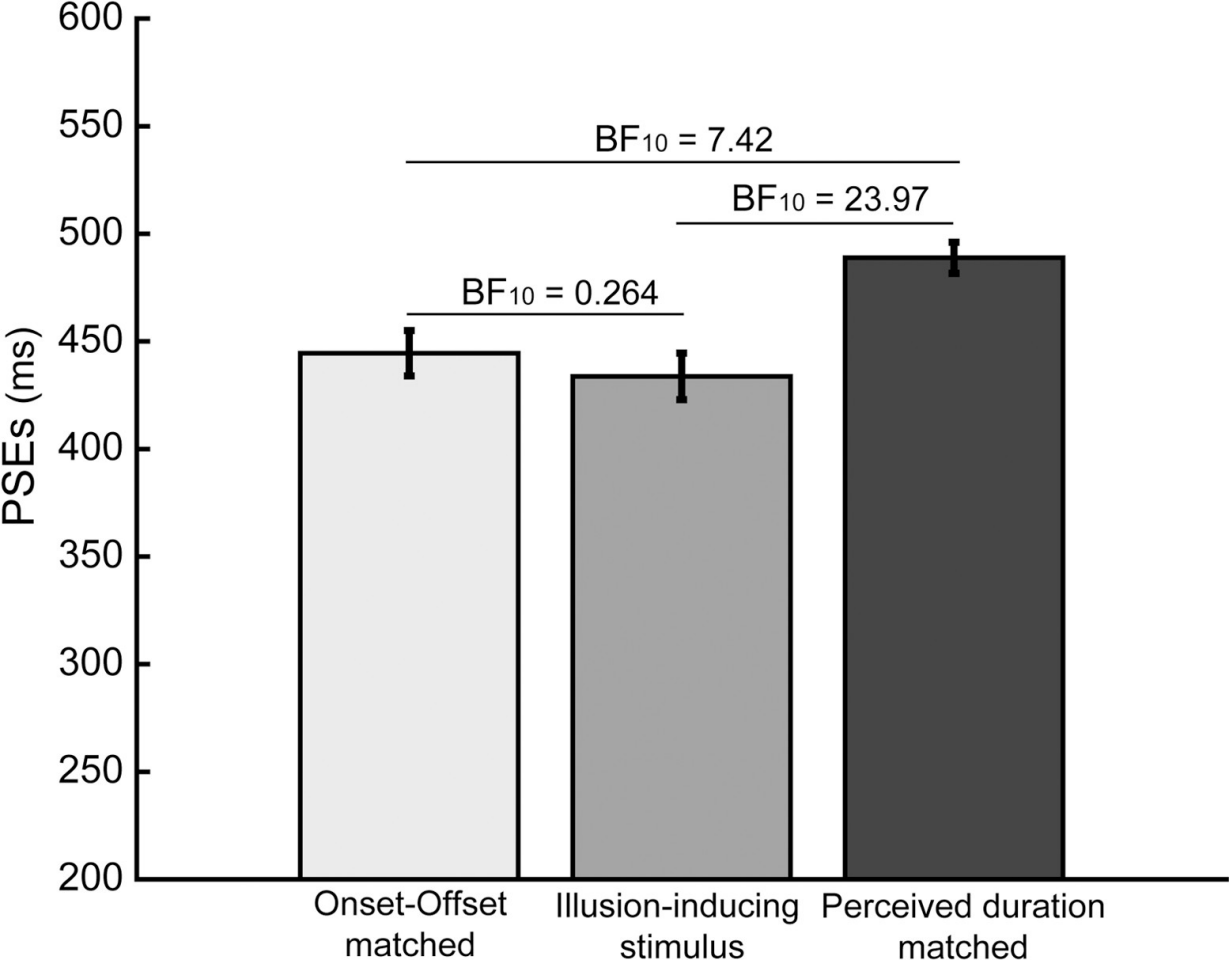
Average PSEs for each of the three adaptation conditions. Average PSEs for when participants adapted to the onset-offset matched stimulus (300 ms, static), the illusion-inducing stimulus (300 ms, 8.33Hz), and the perceptually matched stimulus (M = 572.60 ms, static). BFs are given for all Bayesian paired sample t-tests with BF_10_ > 3 indicating evidence that the PSEs are different and BF_10_ < 1/3 indicating evidence that they are not. Error bars reflect confidence intervals based on the within-subject variability of the data [33,34].

Average PSEs for each of the adaptation conditions can be found in Fig 2. The depicted error bars represent within-subject standard errors calculated using per-subject normalization of the data [33,34]. Error bars depicting the standard of the mean illustrate both the between-subject and within-subject variance in a dataset. These depictions describe the total variance in the data and can help indicated the variability across subjects. However, in a design focused on within-subject differences, error-bars depicting the standard error of the mean have little informational value about the outcome of the within-subject analyses [33]. The within-subject standard errors used here reflect only the within-subject variability making them more predictive of the outcome of the analyses of the within-subject effects that were reported here.

The PSE data were analyzed using a Bayesian repeated measures ANOVA with PSE as a dependent measure and Adaptation Type (onset-offset matched stimulus, illusion-inducing stimulus, perceptually matched stimulus) as a factor. This analysis revealed a main effect of Adaptation type (BF_10_ = 8.14). To gain insight into this main effect we conducted three subsequent pairwise comparisons (Bayesian paired samples t-test) comparing each combination of the adaptation conditions.

First, we compared PSEs between the onset-offset matched stimulus and the perceptually matched stimulus. We found that adaptation to the onset-offset matched duration lead to a longer perceived duration of the test stimulus (M = 444.51 ms, SD = 133.11) compared to adaptation to the perceptually matched duration (M = 488.86 ms, SD = 149.90; BF_10_ = 7.42). In other words, adapting to a static stimulus which lasted 300 ms (onset-offset matched stimulus) caused subsequent test durations to be perceived as having a longer duration, compared to adaptation to a static stimulus with a longer duration (M = 572.60 ms; perceptually matched stimulus). This finding replicates the DAE observed in earlier studies and demonstrates that our method can be used to dissociate adaptation to the durations of the onset-offset matched and perceived duration matched stimuli.

Next, we compared the DAE following adaptation to the illusion-inducing stimulus (M = 433.72 ms, SD = 160.70) to each of the matched conditions and found that the resulting DAE differed from the DAE for the perceptually matched duration (BF_10_ = 23.97), but not from the DAE for the onset-offset matched duration (BF_10_ = 0.264). This suggests that participants adapted to the onset-offset duration of the illusion-inducing stimulus instead of to a duration corresponding to the perceived duration of the illusion-inducing stimulus.

## Discussion

In this study we addressed the apparent contradiction between the proposal that duration-tuned channels encode duration based on the time between the responses to the onset and offset of an event, and the fact that our perception of duration can be dissociated from this onset-offset duration of an event. To this end, we adapted participants to an illusion-inducing stimulus that is known to cause shifts in the perceived duration of an event, without affecting its perceived onset and offset [24]. Participants adapted to one of three types of stimuli: an illusion-inducing rotating radial grating, a static grating matched to the onset-offset duration of the illusion-inducing stimulus, and a static grating matched to the perceived duration of the illusion-inducing stimulus. We measured the resulting DAE and found that the DAE for illusion-inducing stimuli did not differ from the DAE for the onset-offset matched stimuli but did differ from the DAE for the perceptually matched stimuli. In other words, participants adapted to the *onset-offset duration*, and not to a duration corresponding to the *perceived duration* of the illusion-inducing stimulus. This result supports the proposal that duration channels are sensitive to the temporal distance between the onset and offset responses that result from a sensory event; possibly via neurons that show onset-dependent offset responses [1]. We conclude that channel-based duration encoding is based on the temporal distance between the onset and offset of an event and does not necessarily corresponds to the perceived duration of that same event.

Our results demonstrate that duration tuned mechanisms are sensitive to the temporal distance between the onset and offset responses that result from the sensory event that is being encoded. As a result, the duration encoded by these mechanisms does not necessarily correspond to the duration eventually perceived by the observer. This suggest that channel-based encoding reflects an initial processing step, the output of which is then further transformed during subsequent processing. This idea is consistent with a more hierarchical view of duration encoding in which duration information is accumulated from multiple sources across multiple stages of processing [35]. In line with this idea, Heron and colleagues [11] demonstrated that the channel-based encoding of duration occurs before the integration of duration information from the different senses. In addition, several studies have demonstrated that duration perception depends on a wide range of factors that reflect different stages of cognitive processing. For example, studies on memory and memory mixing have demonstrated that memory about other magnitudes can influence estimations of duration [36,37]. Furthermore, studies focusing on the role of contextual experience have demonstrated that duration estimates can be influenced by both the sensory and response history of previous duration estimates [38,39]. In addition to these behavioral findings, a large number of cortical and subcortical areas have been implicated in the processing of duration information (see for example: [10,40–44]). Together, these studies support the notion that duration processing occurs in multiple stages throughout the brain in a distributed and possibly task-specific manner.

Our findings that duration channels do not adapt to a duration corresponding to the perceived duration of our TFITD inducing stimuli draws into question the mechanisms underlying this illusion. Duration illusions are often assumed to reflect direct changes in the encoding of duration (i.e. changing the clock speed), and as such have often been used to study the mechanisms underlying the initial encoding of duration information from sensory information [45–49]. In particular, TFITD has been proposed to reflect changes in the rate at which temporal information is accumulated during duration encoding [45]. In contrast with this assumption, our results suggest that TFITD occurs after the initial (channel-based) encoding of duration and likely reflects modulation of subsequent processing steps. In a more general sense, our finding calls into question the extent to which other duration illusions reflect direct changes in the (rate of) encoding of duration information. That being said, it is important to underscore that only a single illusion was tested in the current study. Since it is likely that different duration illusions influence duration processing in a distinct manner (i.e. at different stages of processing) we should be careful in generalizing the results reported here. In fact, some duration illusions have been reported to be caused by changes in the response to the onset and/or offset of events [50]. Under the current model, these illusions *would* predict changes in the response of the duration-tuned channels and corresponding changes in the duration perceived by the observer. Since most duration illusions are smaller in magnitude then the TFITD illusion employed here, it could prove difficult to apply our paradigm to other duration illusions. Hopefully, future experiments will give insight in the relation between duration-tuned responses and the wide range of duration illusions already reported in the literature.

## References

[1] Heron J, Aaen-Stockdale C, Hotchkiss J, Roach NW, McGraw PV, Whitaker D. Duration channels mediate human time perception. Proc Biol Sci 2012;279:690–8. 10.1098/rspb.2011.1131 21831897 PMC3248727

[2] Ivry RB. The representation of temporal information in perception and motor control. Curr Opin Neurobiol 1996;6:851–7. 10.1016/S0959-4388(96)80037-7 9000026

[3] Hubel DH, Wiesel TN. Receptive fields of single neurones in the cat’s striate cortex. J Physiol (Lond) 1959;148:574–91. 10.1113/jphysiol.1959.sp006308 14403679 PMC1363130

[4] Gibson JJ. Adaptation, after-effect and contrast in the perception of curved lines. J Exp Psychol 1933;16:1–31. 10.1037/h0074626

[5] Albright TD. Direction and orientation selectivity of neurons in visual area MT of the macaque. J Neurophysiol 1984;52:1106–30. 10.1152/jn.1984.52.6.1106 6520628

[6] Anstis S, Verstraten FA, Mather G. The motion aftereffect. Trends Cogn Sci (Regul Ed) 1998;2:111–7. 10.1016/S1364-6613(98)01142-5 21227087

[7] Romani GL, Williamson SJ, Kaufman L. Tonotopic organization of the human auditory cortex. Science 1982;216:1339–40. 10.1126/science.7079770 7079770

[8] Burr D, Ross J. A visual sense of number. Curr Biol 2008;18:425–8. 10.1016/j.cub.2008.02.052 18342507

[9] Harvey BM, Klein BP, Petridou N, Dumoulin SO. Topographic representation of numerosity in the human parietal cortex. Science 2013;341:1123–6. 10.1126/science.1239052 24009396

[10] Hayashi MJ, Ditye T, Harada T, Hashiguchi M, Sadato N, Carlson S, et al Time adaptation shows duration selectivity in the human parietal cortex. PLoS Biol 2015;13:e1002262 10.1371/journal.pbio.1002262 26378440 PMC4574920

[11] Heron J, Hotchkiss J, Aaen-Stockdale C, Roach NW, Whitaker D. A neural hierarchy for illusions of time: duration adaptation precedes multisensory integration. J Vis 2013;13:4–4. 10.1167/13.14.4 24306853 PMC3852255

[12] Li B, Yuan X, Huang X. The aftereffect of perceived duration is contingent on auditory frequency but not visual orientation. Sci Rep 2015;5:10124 10.1038/srep10124 26054927 PMC4460570

[13] Shima S, Murai Y, Hashimoto Y, Yotsumoto Y. Duration Adaptation Occurs Across the Sub- and Supra-Second Systems. Front Psychol 2016;7:114 10.3389/fpsyg.2016.00114 26903920 PMC4746325

[14] Maarseveen J, Hogendoorn H, Verstraten FAJ, Paffen CLE. An investigation of the spatial selectivity of the duration after-effect. Vision Res 2017;130:67–75. 10.1016/j.visres.2016.11.003 27876514

[15] Maarseveen J, Hogendoorn H, Verstraten FAJ, Paffen CLE. Attention gates the selective encoding of duration. Sci Rep 2018;8:2522 10.1038/s41598-018-20850-y 29410447 PMC5802729

[16] Kohn A. Visual adaptation: physiology, mechanisms, and functional benefits. J Neurophysiol 2007;97:3155–64. 10.1152/jn.00086.2007 17344377

[17] Bueti D, Buonomano DV. Temporal Perceptual Learning. Timing & Time Perception 2014;2:261–89. 10.1163/22134468-00002023

[18] Schoups A, Vogels R, Qian N, Orban G. Practising orientation identification improves orientation coding in V1 neurons. Nature 2001;412:549–53. 10.1038/35087601 11484056

[19] Ehrlich D, Casseday JH, Covey E. Neural tuning to sound duration in the inferior colliculus of the big brown bat, Eptesicus fuscus. J Neurophysiol 1997;77:2360–72. 10.1152/jn.1997.77.5.2360 9163363

[20] Wu CH, Jen PHS. Echo frequency selectivity of duration-tuned inferior collicular neurons of the big brown bat, Eptesicus fuscus, determined with pulse-echo pairs. Neuroscience 2008;156:1028–38. 10.1016/j.neuroscience.2008.08.039 18804149

[21] He J, Hashikawa T, Ojima H, Kinouchi Y. Temporal integration and duration tuning in the dorsal zone of cat auditory cortex. J Neurosci 1997;17:2615–25. 9065521 10.1523/JNEUROSCI.17-07-02615.1997 PMC6573496

[22] Duysens J, Schaafsma SJ, Orban GA. Cortical off response tuning for stimulus duration. Vision Res 1996;36:3243–51. 10.1016/0042-6989(96)00040-5 8944284

[23] Johnston A, Arnold DH, Nishida S. Spatially localized distortions of event time. Curr Biol 2006;16:472–9. 10.1016/j.cub.2006.01.032 16527741

[24] Kaneko S, Murakami I. Perceived duration of visual motion increases with speed. J Vis 2009;9:14 10.1167/9.7.14 19761329

[25] Zhou B, Yang S, Mao L, Han S. Visual feature processing in the early visual cortex affects duration perception. J Exp Psychol Gen 2014;143:1893–902. 10.1037/a0037294 25000445

[26] Kesten H. Accelerated Stochastic Approximation. Ann Math Statist 1958;29:41–59. 10.1214/aoms/1177706705

[27] Yumoto N, Lu X, Henry TR, Miyachi S, Nambu A, Fukai T, et al A neural correlate of the processing of multi-second time intervals in primate prefrontal cortex. PLoS One 2011;6:e19168 10.1371/journal.pone.0019168 21556372 PMC3083430

[28] Jamieson DG, Petrusic WM. Presentation order effects in duration discrimination. Percept Psychophys 1975;17:197–202. 10.3758/BF03203886

[29] Saunders JA, Backus BT. Perception of surface slant from oriented textures. J Vis 2006;6:882–97. 10.1167/6.9.3 17083282

[30] Marsman M, Wagenmakers E-J. Bayesian benefits with JASP. European Journal of Developmental Psychology 2017;14:545–55. 10.1080/17405629.2016.1259614

[31] Jeffreys H. The theory of probability. Oxford University Press; 1998.

[32] Lee MD, Wagenmakers E-J. Bayesian cognitive modeling: A practical course. Cambridge: Cambridge University Press; 2013 10.1017/CBO9781139087759

[33] Cousineau D. Confidence intervals in within-subject designs: A simpler solution to Loftus and Masson’s method. TQMP 2005;1:42–5. 10.20982/tqmp.01.1.p042

[34] Morey RD. Confidence Intervals from Normalized Data: A correction to Cousineau (2005). TQMP 2008;4:61–4. 10.20982/tqmp.04.2.p061

[35] van Wassenhove V. Minding time in an amodal representational space. Philos Trans R Soc Lond B, Biol Sci 2009;364:1815–30. 10.1098/rstb.2009.0023 19487185 PMC2685822

[36] Cai ZG, Wang R. Numerical magnitude affects temporal memories but not time encoding. PLoS One 2014;9:e83159 10.1371/journal.pone.0083159 24489646 PMC3906001

[37] Rammsayer TH, Verner M. Larger visual stimuli are perceived to last longer from time to time: The internal clock is not affected by nontemporal visual stimulus size. J Vis 2015;15:5–5. 10.1167/15.3.5 25758710

[38] Roach NW, McGraw PV, Whitaker DJ, Heron J. Generalization of prior information for rapid Bayesian time estimation. Proc Natl Acad Sci USA 2017;114:412–7. 10.1073/pnas.1610706114 28007982 PMC5240697

[39] Jazayeri M, Shadlen MN. Temporal context calibrates interval timing. Nat Neurosci 2010;13:1020–6. 10.1038/nn.2590 20581842 PMC2916084

[40] Jantzen KJ, Steinberg FL, Kelso JAS. Functional MRI reveals the existence of modality and coordination-dependent timing networks. Neuroimage 2005;25:1031–42. 10.1016/j.neuroimage.2004.12.029 15850722

[41] Meck WH, Penney TB, Pouthas V. Cortico-striatal representation of time in animals and humans. Curr Opin Neurobiol 2008;18:145–52. 10.1016/j.conb.2008.08.002 18708142

[42] Mello GBM, Soares S, Paton JJ. A scalable population code for time in the striatum. Curr Biol 2015;25:1113–22. 10.1016/j.cub.2015.02.036 25913405

[43] Merchant H, Pérez O, Zarco W, Gámez J. Interval tuning in the primate medial premotor cortex as a general timing mechanism. J Neurosci 2013;33:9082–96. 10.1523/JNEUROSCI.5513-12.2013 23699519 PMC6705035

[44] Spencer RMC, Ivry RB. Cerebellum and Timing In: Manto M, Schmahmann JD, Rossi F, Gruol DL, Koibuchi N, editors. Handbook of the cerebellum and cerebellar disorders, Dordrecht: Springer Netherlands; 2013, p. 1201–19. 10.1007/978-94-007-1333-8_52

[45] Kanai R, Paffen CLE, Hogendoorn H, Verstraten FAJ. Time dilation in dynamic visual display. J Vis 2006;6:1421–30. 10.1167/6.12.8 17209745

[46] Droit-Volet S, Wearden J. Speeding up an internal clock in children? Effects of visual flicker on subjective duration. Q J Exp Psychol B 2002;55:193–211. 10.1080/02724990143000252 12188524

[47] Xuan B, Zhang D, He S, Chen X. Larger stimuli are judged to last longer. J Vis 2007;7:21–5. 10.1167/7.10.2 17997671

[48] Eagleman DM. Human time perception and its illusions. Curr Opin Neurobiol 2008;18:131–6. 10.1016/j.conb.2008.06.002 18639634 PMC2866156

[49] Pariyadath V, Eagleman D. The effect of predictability on subjective duration. PLoS One 2007;2:e1264 10.1371/journal.pone.0001264 18043760 PMC2082074

[50] Kanai R, Watanabe M. Visual onset expands subjective time. Percept Psychophys 2006;68:1113–23. 10.3758/BF03193714 17355036

